# Effects of acute heat stress on protein expression and histone modification in the adrenal gland of male layer-type country chickens

**DOI:** 10.1038/s41598-021-85868-1

**Published:** 2021-03-22

**Authors:** Hao-Teng Zheng, Zi-Xuan Zhuang, Chao-Jung Chen, Hsin-Yi Liao, Hung-Lin Chen, Huang-Chun Hsueh, Chih-Feng Chen, Shuen-Ei Chen, San-Yuan Huang

**Affiliations:** 1 Department of Animal Science, National Chung Hsing University, 145 Xingda Road, Taichung, 40227 Taiwan; 2 Proteomics Core Laboratory, Department of Medical Research, China Medical University Hospital, 2 Yude Road, Taichung, 40447 Taiwan; 3 Graduate Institute of Integrated Medicine, China Medical University, 91 Hsueh–Shih Road, Taichung, 40402 Taiwan; 4 The iEGG and Animal Biotechnology Center, National Chung Hsing University, 145 Xingda Road, Taichung, 40227 Taiwan; 5 Innovation and Development Center of Sustainable Agriculture (IDCSA), National Chung Hsing University, 145 Xingda Road, Taichung, 40227 Taiwan; 6 Research Center for Sustainable Energy and Nanotechnology, National Chung Hsing University, 145 Xingda Road, Taichung, 40227 Taiwan

**Keywords:** Proteomics, Molecular biology

## Abstract

The adrenal gland responds to heat stress by epinephrine and glucocorticoid release to alleviate the adverse effects. This study investigated the effect of acute heat stress on the protein profile and histone modification in the adrenal gland of layer-type country chickens. A total of 192 roosters were subject to acute heat stress and thereafter classified into a resistant or susceptible group according to body temperature change. The iTRAQ analysis identified 80 differentially expressed proteins, in which the resistant group had a higher level of somatostatin and hydroxy-δ-5-steroid dehydrogenase but a lower parathymosin expression in accordance with the change of serum glucocorticoid levels. Histone modification analysis identified 115 histone markers. The susceptible group had a higher level of tri-methylation of histone H3 lysine 27 (H3K27me3) and showed a positive crosstalk with K36me and K37me in the H3 tails. The differential changes of body temperature projected in physiological regulation at the hypothalamus–pituitary–adrenal axis suggest the genetic heterogeneity in basic metabolic rate and efficiency for heat dissipation to acclimate to thermal stress and maintain body temperature homeostasis. The alteration of adrenal H3K27me3 level was associated with the endocrine function of adrenal gland and may contribute to the thermotolerance of chickens.

## Introduction

Modern chicken breeds dissipate considerable body heat and are sensitive to heat stress due to genetic selection for heightened metabolic activity and meat and egg production^1,2^. Heat stress leads to adverse alterations of behavioral, physiological, reproductive, and immunological responses, causing significant reduction in feed intake, body weight gain, egg production, and meat and egg quality^2–7^. Diminished growth, disease susceptibility, and high mortality resulting from heat stress account for a large part of the cost of poultry production throughout the world^8^.

When a behavioral response fails to meet heat loss requirements under a high ambient temperature, the sympathetic–adrenal–medullary axis (SAM axis) and the hypothalamus–pituitary–adrenal axis (HPA axis) are activated to compensate for the thermal imbalance^9^. Catecholamine (e.g., epinephrine and norepinephrine) and glucocorticoid (GC) release from the SAM axis and HPA axis enhance hepatic glycogenolysis and gluconeogenesis to supply more glucose for energy need in heat stress alleviation^10,11^.

Proteomics is a powerful tool for improving genetic selection, and has been applied in exploring the biological mechanisms of different tissues in response to heat stress^12–14^. These studies provide actual biomarkers for the evaluation of health status and stress tolerance in farm animals^15^. Epigenetics, highly dynamic throughout a lifetime, can affect phenotypes by altering gene expression in response to external or internal factors without altering DNA sequences^16,17^. Mechanisms of epigenetic regulation include DNA methylation, histone modification, and RNA interference^16^. Histone posttranslational modifications (HPTMs) can alter the charge state of histones, which in turn regulates chromatin structure remodeling, the access of transcription factors, and the recruitment of specific binding proteins^18^. Accordingly, environmental factors may alter HPTMs dynamically, leading to differential gene expression and translation in the plasticity and acclimatization of phenotypes. The alterations of HPTMs may serve as biomarkers in the complex traits of domestic animals such as growth, egg production and disease resistance involving a variety of uncovered regulators.

Taiwan country chickens (TCCs) exhibit superior thermotolerance to breeds imported into Taiwan^19^. Body temperature change during heat stress is the simplest parameter to evaluate the adaption of chickens under thermal stress^5^. Behavioral responses to heat stress are commonly adaptive in domestic fowls, but their intensity and duration are highly variable among breeds and individuals^3^. Deciphering the genetic basis of thermotolerance in heat-resistant poultry breeds would provide information benefiting commercial chicken production in tropical areas^20^. However, a global functional genomic study for the profile of protein expressions and HPTMs in the adrenal gland of domestic fowls in response to acute heat stress has not yet been conducted. Only two studies have explored HPTMs in chicken erythrocytes through mass spectrometry (MS)^21,22^. Genomic information explains only a part of the phenotypic variance in thermotolerance. A great portion of variance is embedded in the epigenomic alterations. The potential of applying histone modification and protein markers to decipher the thermotolerance is a novel approach to delineate the mechanisms of thermotolerance in chickens. Therefore, the present study aimed to investigate the effect of acute heat stress on protein expression and histone modification by using MS as a basis for delineation of the molecular mechanisms of adrenal response in the thermotolerance of domestic fowls.

## Results

### Effects of acute heat stress on body temperature changes

**Figure 1:**
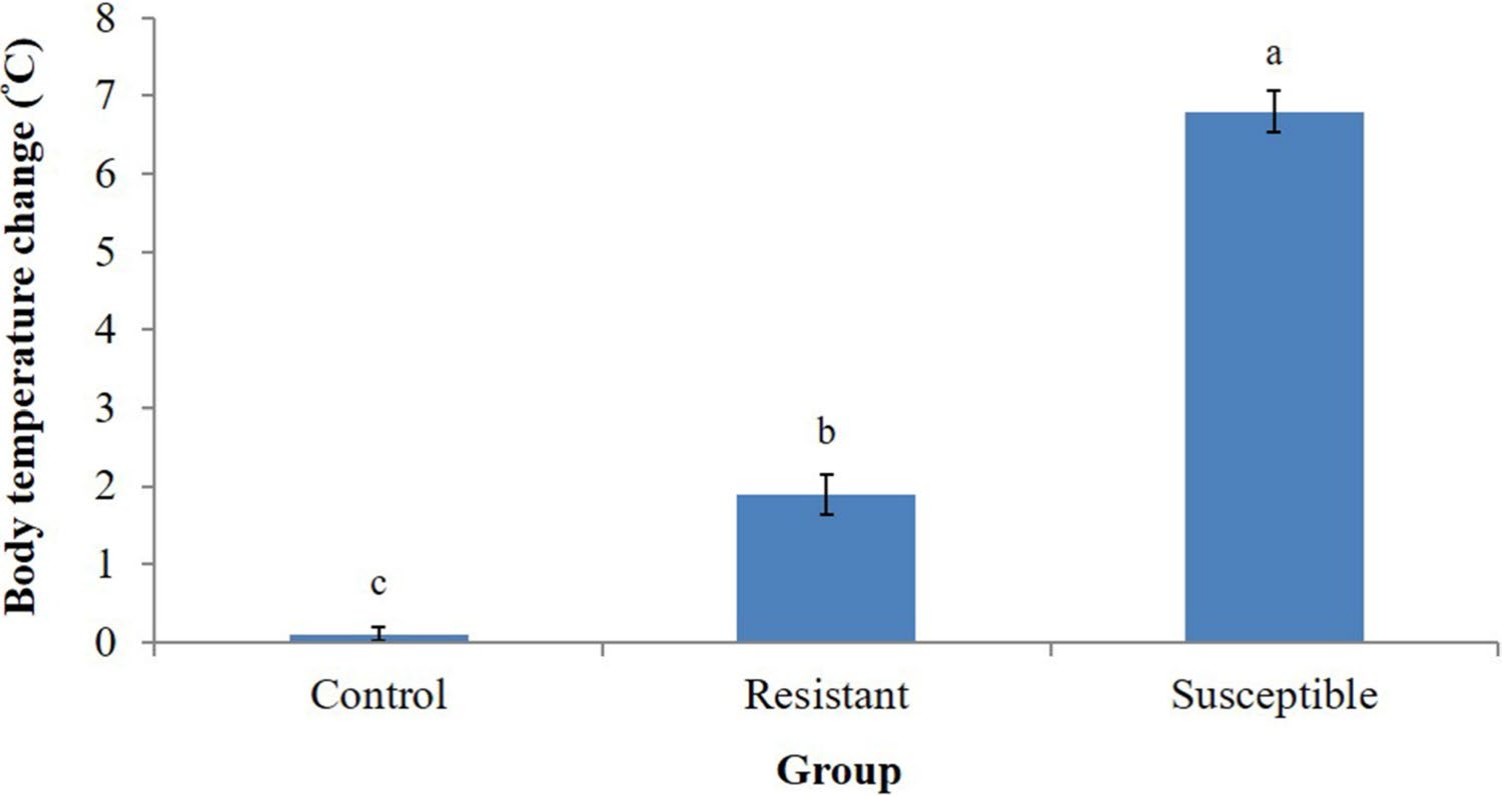
Comparison of body temperature change in male L2 strain Taiwan country chickens (TCCs). ^a,b,c^Least squares means ± standard error (n = 5) with different superscripts indicating a difference between groups (*P* < 0.05).

To group the male layer-type chickens for further analysis of protein expression and histone modification, the change of body temperature after acute heat stress was measured. Heat stress increased the body temperature of resistant and susceptible group. (*P* < 0.05; Fig. 1). In contrast to the susceptible group, the resistant group exhibited a smaller body temperature change after heat stress (*P* < 0.05).

### Protein expression and annotation of differentially expressed proteins (DEPs) in adrenal glands after acute heat stress

**Figure 2:**
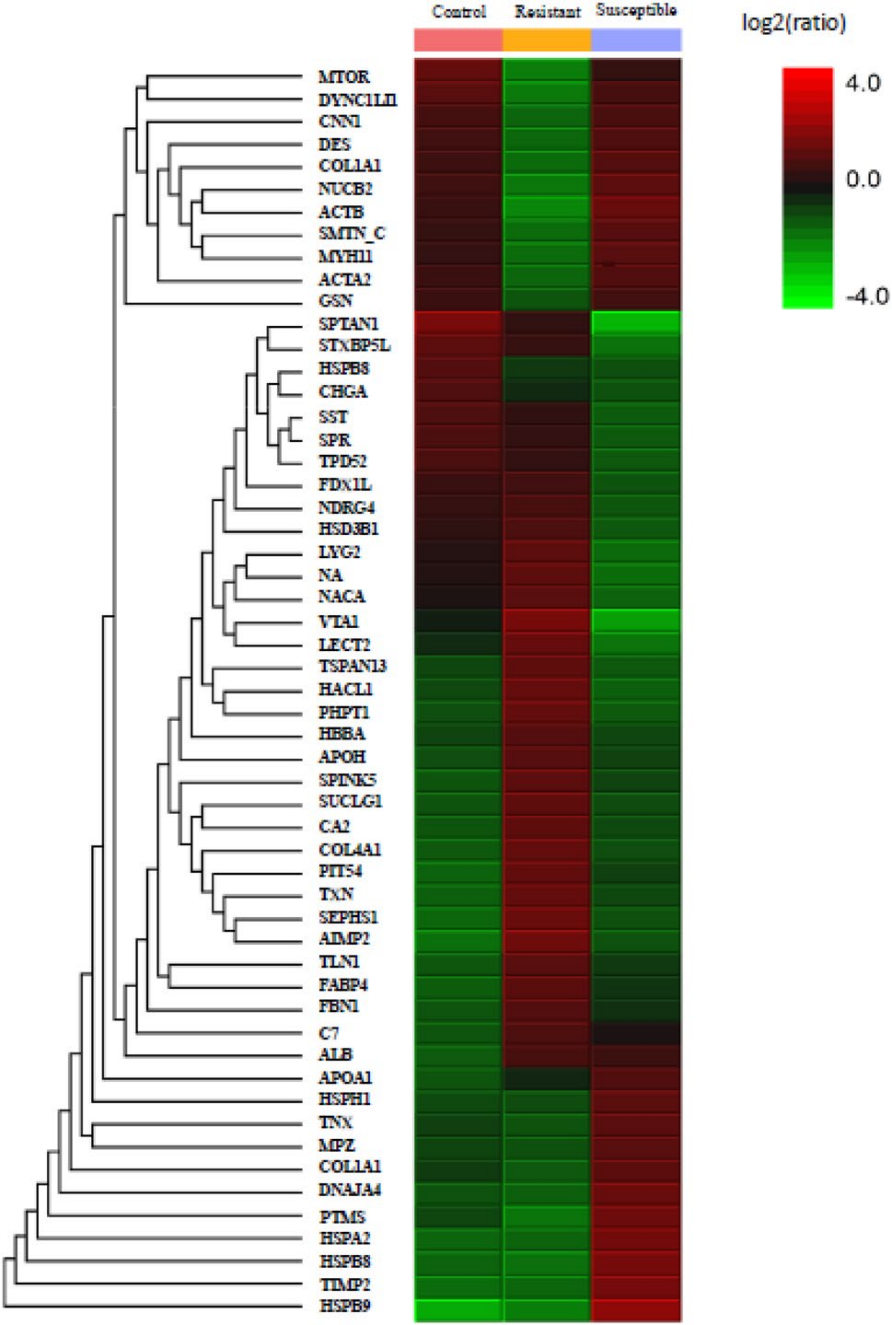
Heat map and hierarchical clustering depicting the differentially expressed proteins in the adrenal gland of male L2 strain Taiwan country chickens after acute heat stress by PEAKS Studio proteomics software (Version X, https://www.bioinfor.com/peaks-studio/). The legend on the right indicates the color key for intermediate ratios.

**Figure 3:**
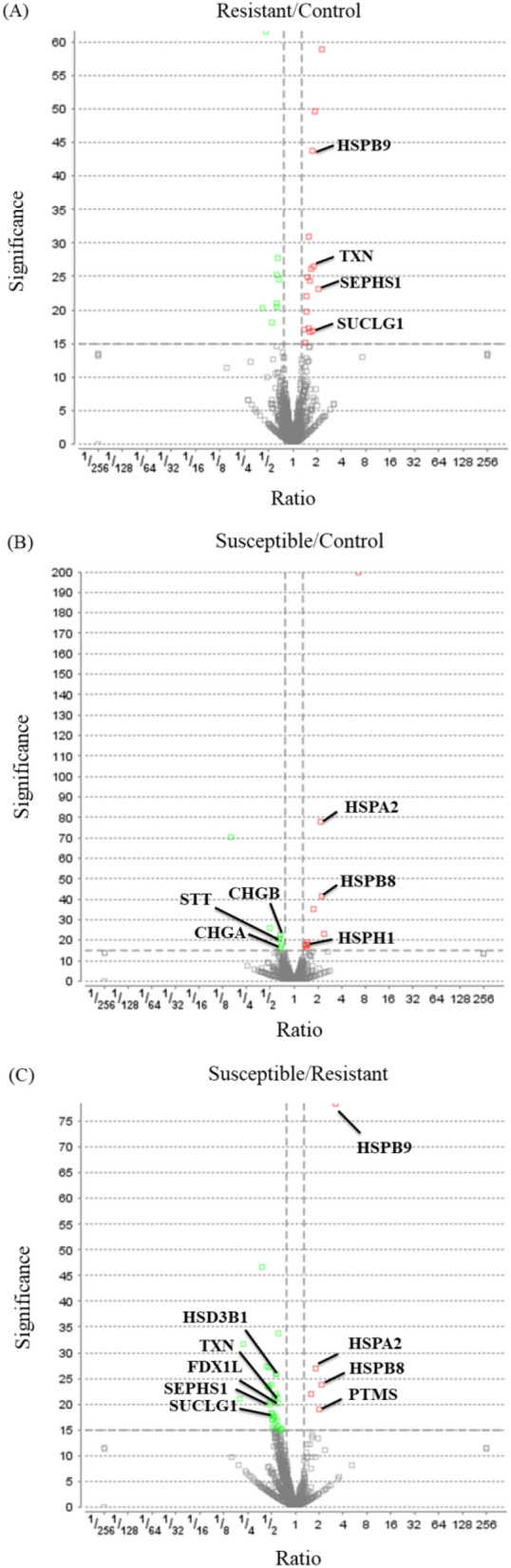
Volcano plots of three pairwise analyses for adrenal glands of male L2 strain TCCs by PEAKS Studio proteomics software (Version X, https://www.bioinfor.com/peaks-studio/) comparing (**A**) Resistant/Control, (**B**) Susceptible/Control, (**C**) Susceptible/Resistant. Comparisons were presented as a statistical significance versus fold-change ratio of protein abundance. Red (upregulated) and green (downregulated) dots represent proteins with significance above 15 and with fold-changes larger than 1.3.

The iTRAQ analysis revealed that 80 of the 5,255 identified proteins were differentially expressed in the heat-stressed groups (Supplementary Table S1). The relatively low numbers of DEP can be attributed to same genetic origin of the strain selected for laying performance for 30 generations. Also, compared to other organs, adrenal gland is a simple tissue with relatively limited functions in physiology and thus may have less DEPs. Gene ontology annotation revealed that most of the DEPs were located in the cell part, intracellular and intracellular organelle (Supplementary Fig. S2). In molecular function, most of the DEPs were mainly categorized by protein binding, ion binding, and organic cyclic compound binding (Supplementary Fig. S2). A heat map demonstrates 80 proteins with significantly differential expression levels in control, resistant, and susceptible groups (Fig. 2). Volcano plots were applied to the data for the identification of most vital DEPs to discriminate between any two groups (Fig. 3). Table 1 shows the 15 most vital DEPs identified in this study.

**Table 1:** Prominent differentially expressed proteins in the adrenal gland of male L2 strain Taiwan country chickens (TCCs) after acute heat stress.

Accession	Description	Gene symbol	Significance	Sequence coverage (%)	Peptides	Unique	Average ratio^a^		
							R/C	S/C	R/S
XP_004935335.1	Secretogranin-1 isoform X1	CHGB	22.57	45	41	41	0.76	0.68	1.12
XP_419377.1	Secretogranin-1 isoform X2	CHGB	22.57	46	41	41	0.76	0.68	1.12
XP_421330.1	Chromogranin-A isoform X1	CHGA	77.7	5	28	3	0.82	0.7	1.17
NP_990667.1	Somatostatin precursor	SST	20.51	42	4	4	0.88	0.65	1.35
AAS66989.1	Somatostatin, partial	SST	20.51	47	4	4	0.88	0.65	1.35
NP_990449.1	Hydroxy-delta-5-steroid dehydrogenase, 3 beta- and steroid delta-isomerase 1	HSD3B1	23.83	54	22	22	1.13	0.76	1.49
XP_015149312.1	Parathymosin	PTMS	30.34	23	3	3	0.71	1.69	0.42
XP_420169.3	Adrenodoxin	FDX1L	16.6	26	8	8	1.04	0.76	1.37
NP_001012910.1	Succinate–CoA ligase [ADP/GDP-forming] subunit alpha, mitochondrial	SUCLG1	16.82	31	6	6	1.61	1.04	1.55
NP_001157556.1	Selenide, water dikinase 1	SEPHS1	23.14	12	3	3	2.06	1.18	1.75
NP_990784.1	Thioredoxin	TXN	26.53	44	6	6	1.83	1.17	1.56
XP_004934466.1	Heat shock protein beta-8	HSPB8	46	16	3	3	0.89	2.24	0.40
NP_001010842.2	Heat shock protein beta-9	HSPB9	200	71	9	8	1.76	6.58	0.27
AAP37959.1	Heat shock protein 70	HSPA2	77.7	42	39	25	1.02	2.19	0.47
NP_001153170.1	Heat shock protein 105 kDa	HSPH1	18.84	23	17	16	0.98	1.48	0.66

*C* control group, *R* resistant group, *S* susceptible group.

^a^Only proteins with a 1.3-fold change for high (> 1.3) or low (< 0.77) relative level, as quantified through iTRAQ, were considered differentially regulated.

### Validation of HSP70 expression in adrenal glands after acute heat stress

**Figure 4:**
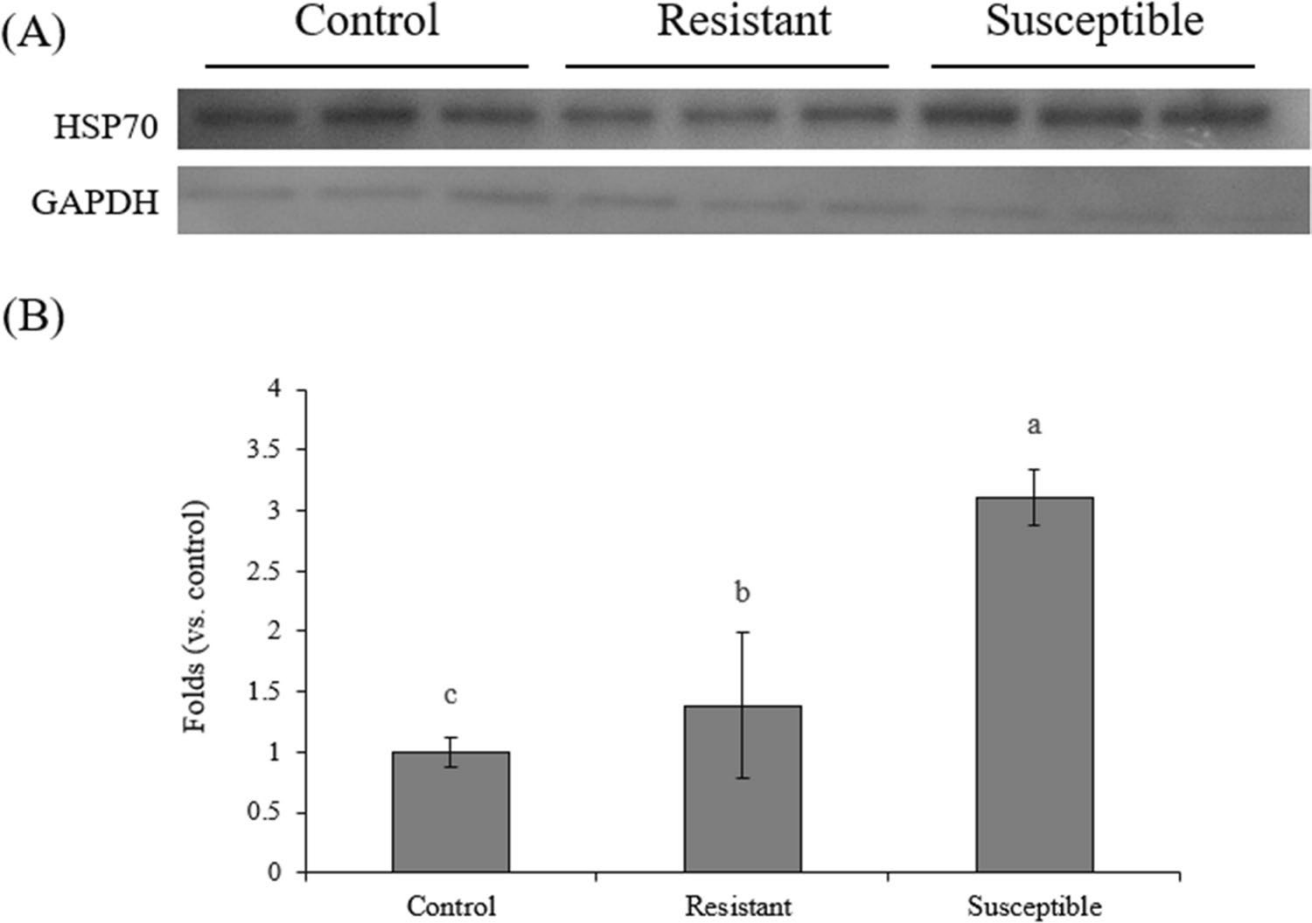
Protein expression levels of HSP70 and GAPDH in the adrenal glands of male L2 strain TCCs. (**A**) A reprehensive Western blot analysis of HSP70 and GAPDH. (**B**) Quantification of relative expression levels of each protein. Results are expressed as mean ± standard error (n = 5). The raw profiles is shown in Supplementary Fig. S3. ^a,b,c^Superscripts indicate a difference between groups (*P* < 0.05).

To validate the DEPs identified by iTRAQ, a Western blot analysis was performed to detect important biochemical indicator HSP70. In contrast to the control, the resistant and susceptible roosters exhibited a higher level of HSP70 (Fig. 4). The level of HSP70 was higher in susceptible roosters than other groups, which was consistent with the results from iTRAQ analysis (Fig. 3 and Table 1).

### Validation of activity of the SAM axis and HPA axis through analysis of plasma adrenaline and CORT concentrations

**Figure 5:**
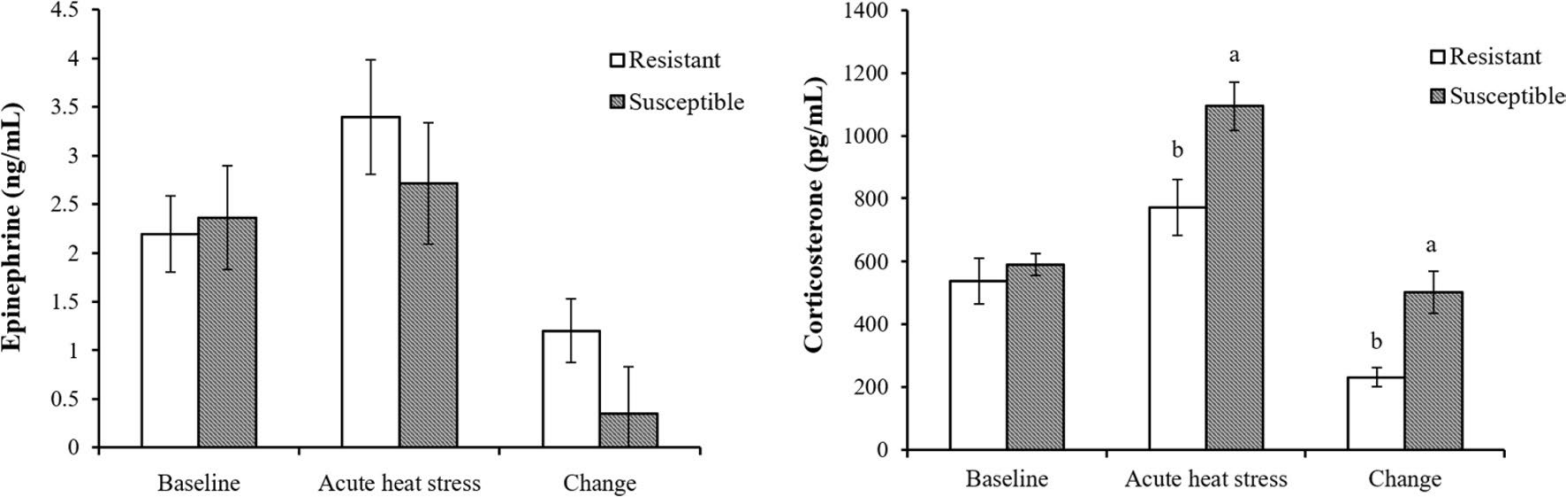
Plasma epinephrine and corticosterone concentrations of male L2 strain TCCs before and after acute heat stress. Results are expressed as mean ± standard error (n = 5). ^a,b^Superscripts indicate a difference between groups (*P* < 0.05).

No differences were discovered in actual plasma epinephrine levels and in their changes after heat stress (Fig. 5). The resistant group exhibited a lower level and change of plasma CORT than did the susceptible group (*P* < 0.05, Fig. 5).

### Acute heat stress modulates adrenal HPTMs

**Figure 6:**
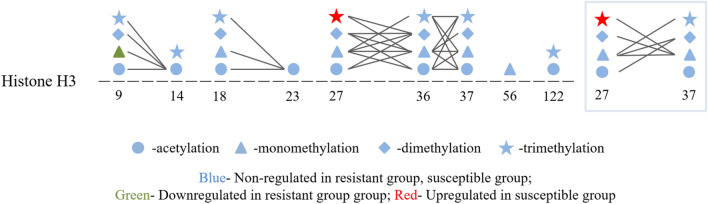
Crosstalks of posttranslational modifications (PTMs) on histone H3 PTMs. Distinctive types of crosstalks are indicated by connecting lines.

**Figure 7:**
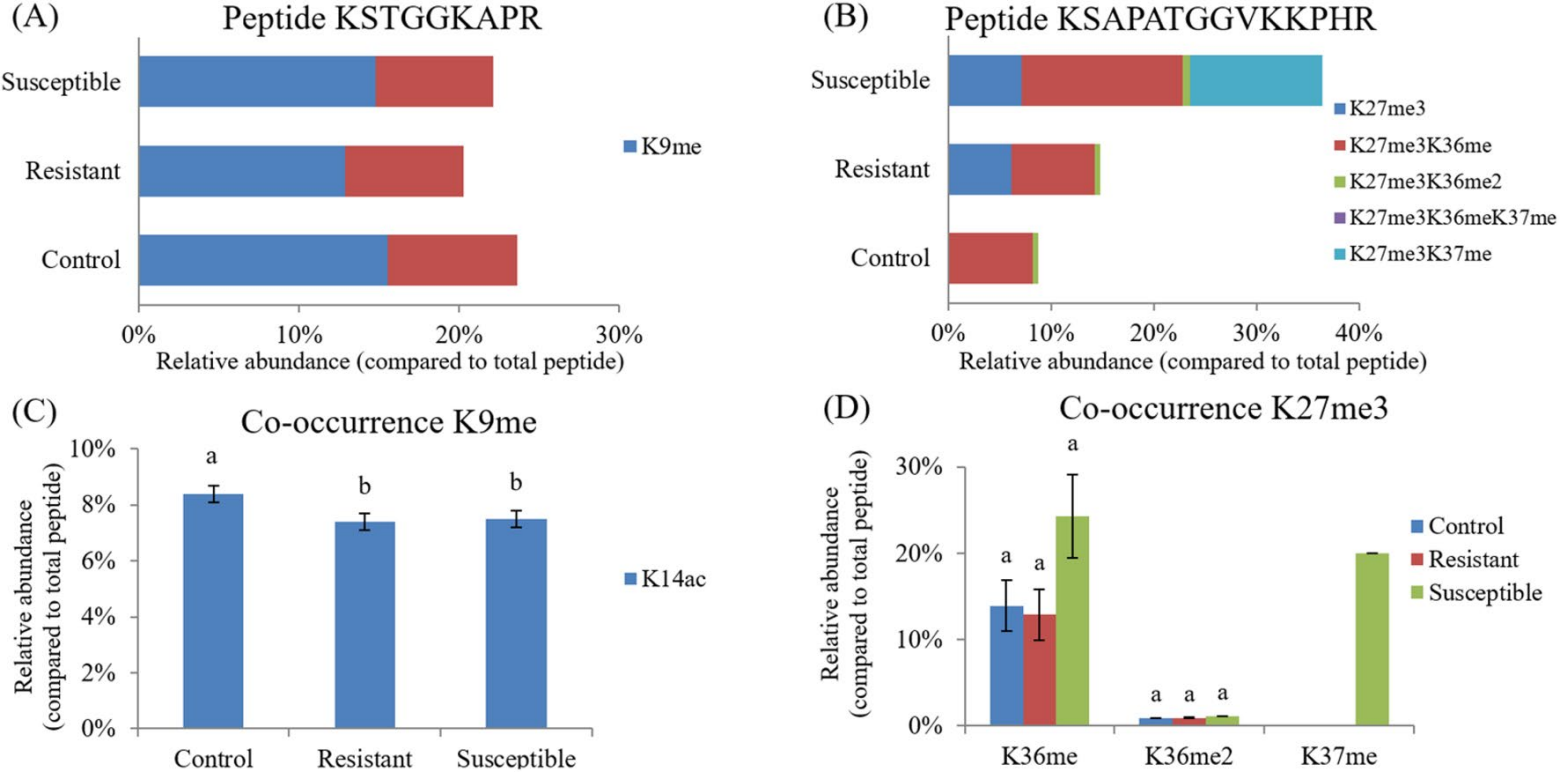
H3K9me and H3K27me3 with distinctive crosstalks that have highly specific histone codes in the adrenal gland of different groups of male L2 strain TCCs. The relative abundance of peptides containing H3K9me and H3K27me3 is depicted in Panels (**A**) and (**B**), respectively, and the relative abundance of PTMs co-expressed with H3K9me and H3K27me3 are shown in Panels (**C**) and (**D**), respectively. Results in Panel (**C**) and (**D**) are expressed as mean ± standard error (n = 5) without missing values. ^a,b^Superscripts indicate a difference between groups within the same PTMs (*P* < 0.05).

The HPTM profile in the adrenal gland of male L2 strain TCCs was analyzed using label-free data-dependent acquisition integrated with LC–MS/MS analysis (Table 2). HPTMs analysis identified 115 histone marks on the N-terminal tails of core and linker histones, including acetylation (ac), monomethylation (me1), dimethylation (me2), and trimethylation (me3). Table 3 provides an overview of the quantified PTMs on histones H3 and H4. In contrast to the control, the resistant roosters exhibited a lower level of H3K9me, and the susceptible group had a higher level of H3K27me3 as compared with the other two groups (*P* < 0.05). The relative quantification of the histone H3 peptide with single- and co-occurring PTMs is illustrated in Supplementary Fig. S4. PTMs with nearby modifications co-occurred in specific combinations and patterns to form specific crosstalks and histone codes (Fig. 6). H3K9me occurred either as a single PTM or in combination with K14ac, whereas H3K27me3 occurred either as a single PTM or in combination with K36me, K36me2, or K37me (Fig. 7A, B). Suppression of the level of H3K9me in the resistant group indicated a negative crosstalk with K14ac, and the K14ac level was lower relative to that in the control group (*P* < 0.05; Fig. 7C). The increased H3K27me3 level in the susceptible group signified a positive crosstalk with K36me and K37me, but their abundance in the three groups did not differ (Fig. 7D).

**Table 2:** Histone posttranslational modifications (PTMs) in the adrenal gland of male L2 strain TCCs.

Histone	Acetylation	Methylation		
		Mono-	Di-	Tri-
H1	K25, K31	K25	K106	K25
H1.01	K25, K31			K25
H1.10	K25, K31			K25
H1.11L	K29, K35			K29
H2AFJ	K5	K9		K5
H2A-IV	K5	K9		K5
H2A-IV-like 3	K5	K9		K5
H2A-IV-like 2	K5	K9		K5
H2bo	K120	K116		K120
H2B-V	K120	K116		K120
H2B-VII	K120	K116		K120
H2B-VIII	K120	K116		K120
H3.3	K9, K14, K18, K23, K27, K36, K37, K79, K122	K18, K23, K27, K36, K37, K56, K79	K9, K18, K27, K36, K37, K79	K18, K27, K36, K37, K79, K122
H3	K9, K14, K18, K23, K27, K36, K37, K122	K9, K18, K23, K27, K36, K37, K56	K9, K18, K27, K36, K37	K9, K14, K18, K27, K36, K37, K122
H4	K16, K31	K31	K20, K31	K31
H5	K12, K14, K119, K120, K132, K133, K135	K12, K119, K120, K132, K133		K119, K120, K133, K135

**Table 3:** Summary of quantified PTMs on histones H3 and H4*.

Histone	Modification	Control	Resistant	Susceptible
H3	K9ac	15.8 ± 1.7	13.7 ± 0.7	15.9 ± 1.2
	K9me	24.5 ± 1.2^a^	20.3 ± 0.8^b^	22.5 ± 0.5^ab^
	K9me2	16.3 ± 3.2	20.6 ± 1.0	16.0 ± 0.6
	K9me3	9.5 ± 0.7	10.8 ± 1.7	9.0 ± 1.9
	K14ac	23.4 ± 1.4	21.3 ± 0.7	22.8 ± 0.7
	K18ac	30.4 ± 1.0	28.7 ± 0.4	30.5 ± 0.5
	K18me	1.3 ± 0.1	1.2 ± 0.1	1.3 ± 0.02
	K23ac	30.8 ± 0.8	29.1 ± 0.8	30.5 ± 0.5
	K23me	0.36 ± 0.02	0.32 ± 0.00	0.35 ± 0.01
	K27ac	8.0 ± 0.0	7.2 ± 4.3	3.7 ± 1.9
	K27me	38.9 ± 5.0	45.1 ± 6.5	36.4 ± 2.8
	K27me2	14.8 ± 6.3	14.9 ± 7.4	10.1 ± 4.5
	K27me3	14.1 ± 3.0^b^	15.2 ± 3.9^b^	30.8 ± 5.7^a^
	K36ac	6.7 ± 3.2	5.0 ± 1.2	7.4 ± 2.4
	K36me	40.7 ± 3.1	44.7 ± 5.5	49.4 ± 3.6
	K36me2	7.9 ± 1.2	8.7 ± 0.4	8.7 ± 0.5
	K36me3	12.6 ± 4.7	11.9 ± 4.3	3.7 ± 1.1
	K37ac	9.1 ± 2.0	14.9 ± 3.2	12.0 ± 0.1
	K37me	16.1 ± 3.2	8.3 ± 2.8	14.3 ± 4.6
	K37me2	0.16 ± 0.00	1.3 ± 0.8	0
	K37me3	10.7 ± 6.4	5.4 ± 0.0	1.8 ± 0.8
	K56me	1.6 ± 0.3	2.1 ± 0.2	12.0 ± 0.1
	K122ac	0.33 ± 0.02	0.32 ± 0.01	0.31 ± 0.02
	K122me3	0.32 ± 0.00	0.34 ± 0.00	0
H4	K31ac	12.4 ± 1.7	9.9 ± 2.8	10.5 ± 0.5
	K31me	7.0 ± 1.6	7.1 ± 3.0	8.2 ± 0.9

*The modification site, type, and relative abundance (%) of each PTM are expressed as the mean ± standard error (n = 5) without missing values. Data were analyzed using the least squares means method and Kruskal–Wallis test.

^a,b^Superscripts indicate a difference between groups within the same PTMs (*P* < 0.05).

## Discussion

Chromogranin A and B (CHGA and CHGB), two major soluble proteins in adrenal medullary chromaffin granules, are implicated in the initiation and regulation of dense core granule (DCG) biogenesis in neuroendocrine cells^23^. CHGA and catecholamines are co-stored in DCGs and released into the circulation^24^. Because serum catecholamine concentration is a poor marker of stress in SAM-axis activity, CHGA is considered as a biomarker of target activation of the SAM axis^25^. A lack of CHGB has been shown to damage DCG biogenesis, leading to dysfunctional adrenal medullary chromaffin granules^23^. Disruption of DCG integrity resulted in insufficient storage of catecholamine^26^. Consistent with these results, downregulation of CHGB and CHGA expression through heat stress (Table 1) may affect DCG biogenesis, leading to decreased storage and secretion of catecholamines and thus impairment of SAM-axis activity.

In response to acute restrain stress, Red Jungle Fowls (RJFs), the primary ancestor of domestic fowls, had a higher HPA axis reactivity accompanied by a quick increase of steroidogenic genes expression and adapted to the stress with a rapid decline of GC secretion than do modern White Leghorn layers^27,28^. Interestingly, growing broilers had a lower basal level of serum GC and exhibited a higher body temperature and change of serum GC levels and HSP expression in response to acute heat stress, whereas higher basal levels of serum GC and no changes of body temperature and HSP expression were observed in RJFs and village chickens of Malaysia^29^. The differences between RJFs and White Leghorns, broilers, and country chickens in HPA axis reactivity to stress reflect the genetic divergence by domestication and selection. Differential protein expressions (Table 1), HPA axis activities (Fig. 2), and body temperature changes (< 2.5 °C vs. > 6.5 °C; Fig. 1) between the resistant and susceptible roosters thus indicate intrinsic heterogeneity in the genetics of the native chickens. Under thermal stress, enhanced SAM axis and HPA axis function to increase glucose supply to meet energy need for systemic heat dissipation^10,11^. A lower activity of HPA axis in the heat-resistant rooster thus may suggests a higher basal metabolic rate and more efficient regulation possibly through sympathetic activity for heat dissipation to maintain body temperature.

The secretion of adrenal GC is regulated in a pulsatile manner through fluctuation of cellular transcript levels of steroidogenic genes including 3β-hydroxysteroid dehydrogenase/δ(5)-δ(4) isomerase type I (HSD3B1) in response to the stimulation of an adrenocorticotropic hormone pulse^30–32^. Because newly synthesized steroidogenic proteins are involved in the rapid synthesis of GC following each pulse of adrenocorticotropic hormone, intracellular stores of steroidogenic proteins are depleted for more pulsatile secretion of GC^31,33^. When the GC level is low, the GR is deactivated by binding with cytosolic chaperones such as heat shock protein 90^34^. Parathymosin functions as a critical coactivator of GR for downstream target gene expression^35,36^. Somatostatin (STT) also plays an inhibitory role in the secretion of multiple hormones and bioactive peptides in the HPA axis^37–39^.

Adrenodoxin promotes iron–sulfur cluster formation of mitochondrial respiratory complexes and thus is involved in adrenal steroidogenesis^40,41^. Succinate-CoA ligase (SUCLG1) regulates mitochondrial DNA synthesis through nucleotide synthesis and transportation for the production of key subunits of mitochondrial respiratory chain complexes^42,43^. Therefore, the resistance to heat stress of roosters may indicate more sufficient mitochondrial function for ATP supply to meet the need for functional adrenal glands for heat dissipation. Greater ATP production thus promotes cellular oxidative stress due to leakage of reactive oxygen species from the mitochondria, leading to upregulation of selenide, water dikinase 1, and thioredoxin in cellular antioxidative defense for achieving cellular redox balance^44–46^. Induction of the HSPs protects cells against the detrimental consequences of heat stress by functioning as molecular chaperones to maintain the normal folding and structure of proteins from further degradation^47^. Therefore, HSPs are important biochemical indicators to evaluate levels of thermal stress^48,49^. In contrast to those of the susceptible group, therefore, upregulation of selenide, water dikinase 1, and thioredoxin and lower HSP expression and HPA activity in the resistant roosters may suggest less oxidative stress by thermal stress in the adrenal gland and sustained function for GC synthesis and secretion, whereas the susceptible birds may progress into adrenal exhaust rapidly due to insufficient ATP supply and oxidative damage^47^.

Cell responses to stimuli depend on the regulation of genetic and epigenetic homeostasis and a dynamic balance of stability and reversibility in gene expression patterns^17^. Therefore, dynamic changes in HPTMs are closely associated with cellular physiological state^18^. Histones H3 and H4 are the best characterized histones and have been suggested to play a prominent regulatory role in chromatin formation^50,51^. H3K9me is involved in transcriptional activation^18^, whereas H3K27me3 is associated with facultative heterochromatin for gene repression^52^. H3K27me3 is among the most studied HPTM^53^, and the present results demonstrated that H3K27me3 is involved in the thermotolerance of male L2 strain TCCs. Previous studies have reported the effect of Marek’s disease on a genome-wide map of H3K27me3 in the immune response of the spleen, thymus, and bursa of Fabricius in chickens^54–56^. Therefore, studies using chromatin immunoprecipitation followed by sequencing (ChIP-seq) technology to investigate adrenal H3K9me and H3K27me3 regulation in specific gene expression and thermotolerance phenotypes are underway.

Histones can be reversibly modified at multiple sites by specific histone-modifying enzymes^57^. Moreover, PTMs have diverse functions and can regulate other PTMs leading to a complex of regulatory crosstalks^58^. A crosstalk can occur on the same histone molecule, between histones, or across nucleosomes; thus, one PTM can affect the occurrence of one or more subsequent PTMs through the interaction of “writers,” “erasers,” and “readers”^58^. The combination of these specific HPTMs through the complex of crosstalk constitutes an epigenetic code that acts as an information-rich signaling platform to recruit downstream “readers” or “effectors” or to directly regulate nucleosomal structure for specific gene transcription and cellular phenotypes^17,59,60^. Epigenetic code is much more significant than the contribution of any single PTM. It elucidates the mechanisms of crosstalk within and between histones in nucleosomes^57^. The length of the peptides generated through propionic anhydride derivatization and trypsin digestion does not give an overview of the entire protein sequence and its modification state, but a crosstalk on the same histone can be observed for nearby modifications. The crosstalk of combinatorial PTMs on adrenal histone H3 (Fig. 6) in this study was specific and may convey distinct biological functions and epigenetic codes. The abundance of H3K9me occurred either as a single PTM or in combination with K14ac, and the abundance of H3K27me3 occurred either as a single PTM or in combination with K36me, K36me2, or K37me (Fig. 7A, B). Co-dependent PTMs within the same histone are typically described as a positive crosstalk, whereas mutually exclusive PTMs are described as a negative crosstalk^61^. The decreased abundance of H3K9me in the resistant roosters indicated a negative crosstalk with K14ac, and the increased abundance of H3K27me3 in susceptible roosters signified a positive crosstalk with K36me and K37me (Fig. 4C, D). Therefore, the interplay of these combinatorial PTMs may play a role in the regulation of the intensity and duration of acute heat stress in the adrenal gland of male L2 strain TCCs and deserves further exploration.

Cell type-specific GR actions have been demonstrated to depend on epigenetics, nucleosome structure, and DNA accessibility^62–64^. Euchromatin, or relaxed chromatin, is transcriptionally active and enriched for active HPTMs (e.g., H3K4me3), whereas heterochromatin or compacted chromatin is transcriptionally repressive and enriched for repressive HPTMs (e.g., H3K27me3)^53^. Therefore, the complex of GC and GR can bind to glucocorticoid response elements and initiate GR-dependent transcription in euchromatin, but it is denied for access to the GC response elements in heterochromatin^62,64^. In contrast to the other groups, the higher level of adrenal H3K27me3 in the susceptible group (Table 3) may indicate a more inaccessible conformation of chromatin remodeling for the access of specific transcription factors.

In summary, heat stress resulted in 80 DEPs in the adrenal glands of male L2 strain TCCs, and histone modification analysis identified 115 PTMs. Functional pathway analysis indicated that the resistant group had a lower activity of HPA axis and HSP expressions but higher mitochondrial function, and antioxidant capacity in the adrenal gland, whereas the susceptible birds exhibited a higher HPA axis activity, but its adrenal chromatin remodeling was constituted mainly in the form of heterochromatin as an increased abundance of H3K27me3. Therefore, dysfunctional maintenance of the homeostasis of body temperature in the susceptible roosters require a stronger stimulation of glucocorticoid for heat dissipation, which ultimately may accelerate the exhaust of adrenal function. The alteration of adrenal H3K27me3 level was associated with adrenal functionality in the thermotolerance of the TCCs. The interplay between HPTMs and phenotypes requires further investigation using technologies such as ChIP-seq.

## Materials and methods

### Management of experimental animals

A flock of the layer-type L2 strain of TCCs, originally bred for egg production by National Chung Hsing University^65^, was reared in the university farm and, at the age of 30 weeks, 197 roosters were used in the study. This study was carried out in compliance with the ARRIVE guidelines (https://arriveguidelines.org). In brief, animal care and use complied with guidelines approved by the Institutional Animal Care and Use Committee (IACUC) of National Chung Hsing University, Taiwan, ROC (IACUC Permit No. 104–112). The roosters were given pellet breeder diet (16.9% crude protein, 3.24% calcium, and 2,930 kcal/kg metabolizable energy) until the end of the experiment. Feed and water were provided ad libitum. Before treatment, the roosters were transferred to individual wire-floored cages in a climate chamber for an adaption period of 2 weeks under the following conditions: a 14:10-h light:dark photoperiod, 25 °C, and 55% relative humidity (RH).

### Conditions of acute heat stress and sample collection

A total of 192 roosters were treated with acute heat stress at 38 °C and 55% RH for 4 h, as described in our previous study^66^. Five roosters were kept at 25 °C and 55% RH as a control group throughout the experiment. No significant differences of body weight among the three groups were observed (Supplementary Fig. S1). All birds were fasted during the heat treatment. Individual body temperature was measured by inserting an alcohol thermometer approximately 2.5 cm into the cloaca before heat treatment and at 0.5, 1, 2, 3, and 4 h into heat treatment. Blood samples were collected from the jugular vein before and after acute heat stress, and plasma was isolated and stored at − 80 °C until hormone analysis. The roosters were grouped by difference in body temperature between the highest value during acute heat stress and value before heat stress. This grouping resulted in definition of a resistant group (ΔT ≤ 2.5 °C) and a susceptible group (ΔT ≥ 6.5 °C). The control group and five roosters from each of the heat-stressed groups were sacrificed for adrenal gland collection^67^ for protein expression and histone modification analysis.

### Plasma epinephrine and corticosterone analysis

Plasma epinephrine and corticosterone (CORT) levels were measured using Adrenaline Research ELISA (BA E-5100, ImmuSmol SAS, Bordeaux, France) and the Corticosterone ELISA Kit (501,320, Cayman Chemical, Ann Arbor, MI, USA), respectively.

### Protein sample preparation, isobaric tags for relative and absolute quantitation (iTRAQ) analysis, and fractionation of peptides

The collected adrenal glands were sliced into small pieces and lysed in O’Farrell’s lysis buffer (9.5 M urea, 65 mM dithiothreitol, 2% v/v Ampholyte 3–10, and 2% NP-40). The samples were sonicated (80 W; four times for 10 s) to dissolute proteins. The homogenates were maintained at 4 °C for 1 h and centrifuged at 14,000 × *g* at 4 °C for 10 min to obtain supernatants. The supernatants were mixed with 100% trichloroacetic acid (TCA) to obtain a final TCA concentration of 20% and maintained at 4 °C for 1 h with shaking every 15 min. After centrifugation at 14,000* g* at 4 °C for 10 min, the precipitated pellets were collected and washed with ice-cold acetone twice. The protein pellets were air-dried for 10 min and dissolved in 4 M urea solution. Protein concentrations were determined using the Bradford method with bovine serum albumin as the standard^68^.

This study performed iTRAQ labeling according to the manufacturer’s protocol (iTRAQ reagent multiplex kit, Applied Biosystems, Waltham, MA, USA). Five replicated protein samples from the same group were mixed and used for reduction and alkylation, which was followed by overnight digestion with trypsin. The tryptic peptides from the control, resistant, and susceptible groups were labeled with isobaric iTRAQ tags with mass 114, 115, and 116 Da, respectively. The samples were then pooled, dried using a SpeedVac evaporator (Tokyo Rikakikai Co. Ltd., Bunkyo-ku, Tokyo, Japan), and stored at − 80 °C until analysis.

Fractionation of the labeled peptides was performed using an ultraperformance liquid chromatography (UPLC) system (ACQUITY UPLC System, Waters, Milford, MA, USA) and a 2.1 mm × 150 mm × 1.7 µm column with a volume of 0.519 mL (ACQUITY UPLC BEH C_18_, Waters). The mobile phase was prepared in a gradient with 10 mM ammonium bicarbonate (ABC, pH 10, mobile phase A) and 10 mM ABC/90% acetonitrile (pH 10, mobile phase B). A gradient was created with mobile phase B from 0 to 3% during min 0–5; 3% to 30% during min 5–40; 30% to 70% during min 40–55; and 70% to 0% during min 55–60. The flow rate was 0.2 µL/min. Fractions were collected in 1-min intervals for 1 h duration. Urea solutions in various fractions were removed using C_18_ ZipTip (Merck, Darmstadt, Germany). All fractions were dried using a SpeedVac evaporator (Tokyo Rikakikai Co. Ltd.) and stored at − 80 °C until analysis.

### Protein identification using nano-UPLC–electrospray ionization (ESI)–quadruple time-of-flight (Q-TOF)–MS/MS

A nano-LC–MS/MS system was used to analyze the tryptic peptides. The peptides were separated using an Ultimate 3000 LC RSLC nano-LC system (Dionex-Thermo Scientific, Chelmsford, MA, USA) coupled with a Q-TOF mass spectrometer (maXis impact, Bruker Daltonics Inc., Bremen, Germany). Each dried fraction was dissolved in 10 µL loading buffer (2% acetonitrile (ACN) and 0.1% FA) and injected into a C18 trapping column (Acclaim PepMap C_18_, Dionex-Thermo Scientific) connected to a C_18_ analyst column (Acclaim PepMap C_18_, Dionex-Thermo Scientific) for peptide separation. The labeled peptides were eluted using a linear gradient of mobile phase A (2% ACN and 0.1% FA) and mobile phase B (80% ACN and 0.1% FA) applied at a flow rate of 0.3 µL/min for 90 min. The gradient conditions were as follows: 5% to 30% mobile phase B during min 5–65; 30% to 98% mobile phase B during min 65–79, and finally, down to 10% mobile phase B within 1 min.

The mass spectrometer was operated at 50–2000 m/z at 2 Hz, and the 20 most intense ions with 420–2000 m/z in each survey scan were selected for the MS/MS experiment. MS/MS data were acquired from 50 to 2000 m/z at 5–10 Hz. The MS/MS spectra were de novo sequenced and assigned a protein ID by using PEAKS X (Version X, ; Bioinformatics Solutions, Waterloo, Canada) and searched against the NCBInr database (NCBInr 20,180,904 version) for protein identification. The false discovery rate (FDR) of peptide identification was set to be less than 1%. Protein quantification was achieved using PEAKS X with a significant score (− 10log*P*) > 15 equal to a *P*-value < 0.03 using FDR-corrected peaks, and at least one unique peptide was detected. Totally, 80 DEPs were identified and quantified using iTRAQ analysis with a 1.3-fold change for a high (> 1.3) or low (< 0.77) level of relative abundance being considered as differentially expressed proteins (DEPs) of the upregulation or downregulation between the two compared groups, respectively. The volcano diagrams and hierarchical clustering of DEPs were generated by PEAKS X software (Bioinformatics Solutions).

### Bioinformatics analysis of DEPs

The DEPs among the groups were annotated for their cellular components, biological processes, and molecular functions by using the Gene Ontology database (amigo1.geneontology.org/cgi-bin/amigo/go.cgi).

### Western blot analysis

In electrophoresis for protein separation, each well contained a respective sample with 50 µg of proteins. Proteins were transferred onto PVDF (polyvinylidene fluoride) membrane through the wet-transfer method. A mouse anti-HSP70 (clone N27F3-4) monoclonal antibody was purchased from Enzo Life Sciences (New York, USA). A mouse anti-GAPDH (clone 1D4) monoclonal antibody was purchased from Novus Biologicals (Denver, USA). Horseradish peroxidase conjugated secondary antibodies; goat anti-mouse IgG (Beckman Coulter, Brea, CA, USA) was used for to identify the bands reactive to the primary antibodies through an enhanced chemiluminescence reagent (Pierce Biotechnology Inc., Rockford, IL, USA). Primary and secondary antibodies were incubated with membranes at 1:1000 and 1:5000 dilation, respectively. Signaling was quantified by the luminescence image analyzer ImageQuant LAS 4000 (GE Healthcare Life Sciences).

### Histone sample preparation, chemical derivatization, trypsin digestion, and desalting

Histones were isolated using a modified protocol^69^. Briefly, nuclei were isolated with nuclei isolation buffer (NIB; 15 mM Tris, 60 mM KCl, 15 mM NaCl, 5 mM MgCl_2_, 1 mM CaCl_2_, and 250 mM sucrose and protease inhibitor cocktail tablet; pH 7.5) and 0.2% NP-40. After they had been cut into small pieces, the adrenal glands in NIB were homogenized using a homogenizer (T 10 basic ULTRA-TURRAX, IKA, Guangzhou, China), which was followed by 10 min incubation on ice. The mixture was centrifuged at 1,000 × *g* at 4 °C for 10 min, and the resultant nuclei pellets were collected. The pellets were washed with NIB twice. Histones were then acid-extracted from the isolated nuclei by using 0.2 M H_2_SO_4_ at 4 °C for 4 h with shaking every 15 min. The histone-containing supernatants were mixed with 100% TCA to a final TCA concentration of 33% and incubated on ice for 1 h. The histone-enriched pellets were washed with ice-cold acetone/0.1% hydrochloric acid and ice-cold acetone and centrifuged to enable pellet collection. The collected pellets were air-dried and reconstituted in double-distilled water. Finally, the histones were purified through centrifugation and quantified for concentration by using the Bradford method with bovine serum albumin as the standard (Peterson, 1983). All samples were dried using a SpeedVac evaporator (Tokyo Rikakikai Co. Ltd.) and dissolved in 40 μL of 50 mM ammonium bicarbonate, which had pH 8 (concentration > 1 μg/μL). Histones were prepared for MS analysis through propionic anhydride chemical derivatization, trypsin digestion, and propionylation of histone peptides at N-termini, as was described by Sidoli et al. (2016). Then, all histone peptides were desalted with C_18_ ZipTip (Merck), dried using the SpeedVac evaporator, and finally stored at − 80 °C until analysis.

### Identification of histone modifications by using nano-UPLC-ESI-Q-TOF–MS/MS

Nano-LC–MS/MS and the protocol for identification of histone modifications were performed as is described in “Acute heat stress modulates adrenal HPTMs” section. Briefly, histone peptides dissolved in 10 µL of loading buffer were separated and eluted using a linear gradient of mobile phase A (2% ACN, 0.1% FA) and mobile phase B (80% ACN, 0.1% FA) applied at a flow rate of 0.3 µL/min for 90 min. The gradient conditions were as follows: 10% to 40% mobile phase B at min 6–74, 40% to 99% mobile phase B at min 74.1–79, and finally, down to 10% mobile phase B within 1 min.

The MS parameters were as described in “Acute heat stress modulates adrenal HPTMs” section. Label-free quantification was performed using the quantitation module of PEAKS X. Modified histone peptides were identified using PEAKS X through the following search parameters: parent mass error tolerance: 80.0 ppm; fragment mass error tolerance: 0.07 Da; enzyme: trypsin; maximum number of missed cleavages: 2; digestion mode: specific; fixed modifications: propionyl (N-term): 56.0; variable modifications: oxidation (M): 15.99, acetylation (K): 42.01, dimethylation (K): 28.03, methylation (K): 14.02, trimethylation (K): 42.05, propionyl (K): 56.03, deamidation (NQ): 0.98, propionylmethyl: 70.04; maximum number of variable PTMs per peptide: 9; reported number of peptides: 5; and data refine dependencies: 1, 4, 3, 2, 5, 6, 7, 8, 9, 10, 11, 12, 14, 13, 15, 16, 17, 19, 18, and 20. The quantification of histone modification was performed using the PEAKS DB database, which provided an overview of all peptides and histone modifications. The relative abundance of a given PTM resulting from single- or co-occurring PTMs was calculated by dividing its intensity by the sum of intensities for all modified and unmodified peptides sharing the same sequence and without missing values. Therefore, the given PTMs could have only a single datum. The quantification of each peptide of co-occurring PTMs on histone H3 was divided by the quantification of all modified and unmodified peptides to obtain a relative quantification of the histone H3 peptide and the crosstalk of PTMs on histone H3.

### Statistical analysis

The concentrations of plasma epinephrine and CORT were analyzed using Student’s *t* test in the Statistical Analysis System (SAS) software^70^. The normality of the body temperature changes, western blot analysis and relative values of DEPs and HPTMs were assessed using the normality test. Normally distributed data were analyzed using the least squares means procedure, whereas non-normally distributed data were analyzed using the Kruskal–Wallis test.

## Supplementary Information

Supplementary Information 1.
