# Supplementary material for: Effects of acute heat stress on protein expression and histone modification in the adrenal gland of male layer-type country chickens
Source: Sci Rep. 2021 Mar 22;11:6499. doi: 10.1038/s41598-021-85868-1 (PMC7985386; doi:10.1038/s41598-021-85868-1)
Supplement: Supplementary file 1 — Supplementary Information 1. [file 41598_2021_85868_MOESM1_ESM.pdf]

Effect of acute heat stress on protein expression and histone modification in the adrenal gland of male layer-type country chickens

Hao-Teng Zheng[1], Zi-Xuan Zhuang[1], Chao-Jung Chen[2,3], Hsin-Yi Liao[2], Hung-Lin Chen[1], Huang-Chun Hsueh[1], Chih-Feng Chen[1,4], Shuen-Ei Chen[1,4,5,6], and San-Yuan Huang[1,4,,6]

Supplementary information

Supplementary Table S1. Differentially expressed proteins in the adrenal gland of male L2 strain Taiwan country chickens (TCCs) after acute heat stress.

Accession	Description	Gene symbol	Significance	Coverage (%)	Peptides	Unique	Average ratio[a]		
							R/C	S/C	R/S
P10587.4	Myosin-11	MYH11	51.02	44	112	88	0.65	1.2	0.54
NP_990605.2	Myosin-11	MYH11	51.02	44	112	88	0.65	1.2	0.54
NP_001036003.1	Spectrin alpha chain, non-erythrocytic 1	SPTAN1	70.55	1	119	2	0.62	0.16	3.88
XP_015132714.1	Talin-1 isoform X1	TLN1	17.32	2	95	3	1.58	1.16	1.36
NP_990592.2	Serum albumin precursor	ALB	22	82	53	53	1.46	1.38	1.06
XP_015147420.1	Fibrillin-1 isoform X1	FBN1	24.82	28	64	64	1.5	1.18	1.27
XP_004935335.1	Secretogranin-1 isoform X1	CHGB	22.57	45	41	41	0.76	0.68	1.12
XP_419377.1	Secretogranin-1 isoform X2	CHGB	22.57	46	41	41	0.76	0.68	1.12
AAP37959.1	Heat shock protein 70	HSPA2	77.7	42	39	25	1.02	2.19	0.47
XP_421330.1	Chromogranin-A isoform X1	CHGA	17.97	5	28	3	0.82	0.7	1.17
P02542.1	Desmin	DES	34.25	9	39	3	0.62	1.13	0.55
BAA25132.1	Desmin, partial	DES	25.23	16	42	6	0.64	1.08	0.59
NP_001026400.1	Actin, aortic smooth muscle	ACTA2	37.41	47	26	12	0.67	1.12	0.60
NP_990265.1	Gelsolin precursor	GSN	16.14	35	22	22	0.76	1.03	0.74
NP_990449.1	Hydroxy-delta-5-steroid dehydrogenase, 3 beta- and steroid delta-isomerase 1	HSD3B1	23.83	54	22	22	1.13	0.76	1.49
NP_001025783.2	Ovoinhibitor precursor	SPINK5	30.95	39	16	16	1.59	1.11	1.43
NP_990820.1	Hemoglobin subunit beta	HBBA	18.29	82	15	15	1.38	0.98	1.41
NP_997063.1	PIT54 protein precursor	PIT54	49.59	40	16	16	1.84	1.23	1.50
CAA28501.1	Carbonic anhydrase 2	CA2	24.58	62	12	12	1.62	1.07	1.51
NP_990648.1	Carbonic anhydrase 2	CA2	24.58	61	12	12	1.62	1.07	1.51
NP_001305331.1	Complement component C7 precursor	C7	15.1	27	17	17	1.44	1.23	1.17

NP_001153170.1	Heat shock protein 105 kDa	HSPH1	18.84	23	17	16	0.98	1.48	0.66
CAA67509.1	Tenascin Y variant	TNX	17.83	9	11	11	0.9	1.4	0.64
BAF63010.1	Tenascin X B	TNX	17.83	8	11	11	0.9	1.4	0.64
NP_001010842.2	Heat shock protein beta-9	HSPB9	200	71	9	8	1.76	6.58	0.27
NP_001264923.1	Beta-2-glycoprotein 1 precursor	APOH	19.73	37	10	10	1.47	1.06	1.39
P26932.2	Calponin-1	CNN1	22.92	58	16	15	0.64	1.03	0.62
NP_001161209.1	Cytoplasmic dynein 1 light intermediate chain 1	DYNC1LI1	62.01	28	9	9	0.47	0.89	0.53
XP_015136979.1	Cytoplasmic dynein 1 light intermediate chain 1 isoform X1	DYNC1LI1	62.01	26	9	9	0.47	0.89	0.53
NP_990809.2	Myeloid protein 1 precursor	LECT2	46.44	48	11	11	1.43	0.65	2.20
P08940.2	Myeloid protein 1	LECT2	46.44	48	11	11	1.43	0.65	2.20
XP_413746.5	DnaJ homolog subfamily A member 4 isoform X1	DNAJA4	37.62	29	8	8	0.92	1.74	0.53
P02457.3	Collagen alpha-1(I) chain	COL1A1	33.93	10	11	10	0.85	1.39	0.61
XP_420169.3	Adrenodoxin	FDX1L	16.6	26	8	8	1.04	0.76	1.37
NP_990856.1	Apolipoprotein A-I preproprotein	APOA1	17.8	52	15	15	1.22	1.49	0.82
AAA48597.1	Apolipoprotein A-I	APOA1	17.8	52	15	15	1.22	1.49	0.82
XP_004944071.1	Protein NDRG4 isoform X5	NDRG4	15.41	24	5	5	1.08	0.76	1.42
NP_989621.1	Fatty acid-binding protein, adipocyte	FABP4	26.22	35	6	5	1.67	1.23	1.36
NP_001073222.1	Tumor protein D52	TPD52	16.6	51	6	6	0.9	0.68	1.32
XP_015138320.1	Tumor protein D52 isoform X7	TPD52	16.6	48	6	6	0.9	0.68	1.32
XP_015138319.1	Tumor protein D52 isoform X6	TPD52	16.6	47	6	6	0.9	0.68	1.32
XP_015138321.1	Tumor protein D52 isoform X5	TPD52	16.6	45	6	6	0.9	0.68	1.32
XP_015138316.1	Tumor protein D52 isoform X4	TPD52	16.6	43	6	6	0.9	0.68	1.32
XP_015138311.1	Tumor protein D52 isoform X1	TPD52	16.6	39	6	6	0.9	0.68	1.32

NP_001012910.1	Succinate--CoA ligase [ADP/GDP-forming] subunit alpha, mitochondrial	SUCLG1	16.82	31	6	6	1.61	1.04	1.55
XP_015149769.1	Aminoacyl tRNA synthase complex-interacting multifunctional protein 2 isoform X1	AIMP2	59.18	22	4	4	2.3	1.25	1.84
P37301.1	Myelin protein P0	MPZ	19.89	22	7	7	0.93	1.43	0.65
NP_001345858.1	Myelin protein P0 isoform MPZ precursor	MPZ	19.89	22	7	7	0.93	1.43	0.65
NP_001345859.1	Myelin protein P0 isoform L-MPZ precursor	MPZ	19.89	18	7	7	0.93	1.43	0.65
NP_990667.1	Somatostatin precursor	SST	20.51	42	4	4	0.88	0.65	1.35
AAS66989.1	Somatostatin, partial	SST	20.51	47	4	4	0.88	0.65	1.35
NP_001186557.1	2-hydroxyacyl-CoA lyase 1	HACL1	21.67	13	5	5	1.58	0.86	1.84
XP_015133310.1	Collagen alpha-1(IV) chain isoform X2	COL4A1	17.03	5	6	4	1.74	1.08	1.61
XP_015133309.1	Collagen alpha-1(IV) chain isoform X1	COL4A1	17.03	5	6	4	1.74	1.08	1.61
NP_001155871.1	Collagen alpha-1(IV) chain precursor	COL4A1	17.03	5	6	4	1.74	1.08	1.61
AEP68181.1	Beta-actin, partial	ACTB	16.37	12	4	1	0.48	1.32	0.36
NP_001263232.1	Nascent polypeptide-associated complex subunit alpha	NACA	21.3	27	4	4	1.26	0.73	1.73
XP_423038.1	Sepiapterin reductase	SPR	15.54	28	5	5	0.9	0.67	1.34
XP_015135021.1	14 kDa phosphohistidine phosphatase isoform X1	PHPT1	17.81	28	4	4	1.63	0.93	1.75
AAF81786.1	Smoothelin-C	SMTN_C	18.5	23	8	8	0.65	1.19	0.55
XP_015150541.1	Syntaxin-binding protein 5-like isoform X7	STXBP5L	21.61	7	4	3	0.81	0.49	1.65
XP_015150523.1	Syntaxin-binding protein 5-like isoform X5	STXBP5L	21.61	7	4	3	0.81	0.49	1.65

XP_015150601.1	Syntaxin-binding protein 5-like isoform X16	STXBP5L	21.61	7	4	3	0.81	0.49	1.65
XP_015150596.1	Syntaxin-binding protein 5-like isoform X15	STXBP5L	21.61	7	4	3	0.81	0.49	1.65
XP_015150569.1	Syntaxin-binding protein 5-like isoform X10	STXBP5L	21.61	7	4	3	0.81	0.49	1.65
NP_990784.1	Thioredoxin	TXN	26.53	44	6	6	1.83	1.17	1.56
ABF46812.1	LH beta subunit, partial	NA	27.33	55	3	3	1.3	0.67	1.94
ADY03193.1	LH beta subunit	NA	27.33	25	3	3	1.3	0.67	1.94
XP_004934466.1	Heat shock protein beta-8	HSPB8	46	16	3	3	0.89	2.24	0.40
XP_015155849.1	Coatomer subunit zeta-1 isoform X2	COPZ1	18.3	23	3	3	0.56	1.18	0.47
NP_001001470.1	Lysozyme g precursor	LYG2	19.05	24	4	4	1.28	0.68	1.88
XP_015133176.1	Lysozyme g isoform X1	LYG2	19.05	24	4	4	1.28	0.68	1.88
NP_001157556.1	Selenide, water dikinase 1	SEPHS1	23.14	12	3	3	2.06	1.18	1.75
XP_015136896.1	Tetraspanin-13 isoform X1	TSPAN13	15.11	15	3	3	1.51	0.9	1.68
NP_001186541.1	Tetraspanin-13 [Gallus gallus]	TSPAN13	15.11	14	3	3	1.51	0.9	1.68
XP_015139683.1	Vacuolar protein sorting-associated protein VTA1 homolog isoform X2	VTA1	20.24	15	2	2	1.63	0.38	4.29
XP_419713.4	Vacuolar protein sorting-associated protein VTA1 homolog isoform X1	VTA1	20.24	13	2	2	1.63	0.38	4.29
NP_989629.1	Metalloproteinase inhibitor 2 precursor	TIMP2	22.93	18	4	4	1.05	2.4	0.44
XP_015149312.1	Parathymosin	PTMS	30.34	23	3	3	0.71	1.69	0.42
XP_417614.3	Serine/threonine-protein kinase mTOR	MTOR	20.39	2	3	3	0.42	0.76	0.55

[a] Only proteins with 1.3-fold change for high (>1.3) or low (< 0.77) relative protein level, as quantified through iTRAQ, were considered differentially regulated.

C, control group; R, resistant group; S, susceptible group.

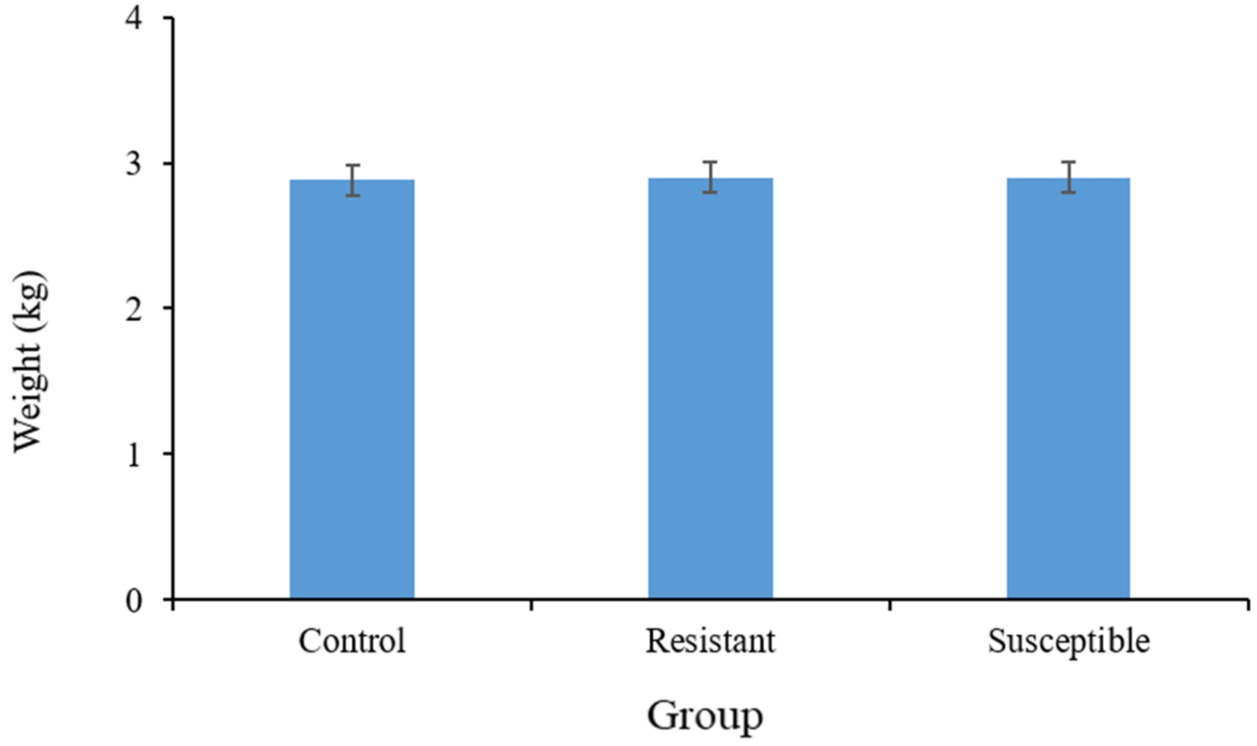

Supplementary Fig. S1. Comparison of body weight in male L2 strain Taiwan country chickens.
Least squares means ± standard error (n = 5) were presented. No significant differences among the three groups were observed.

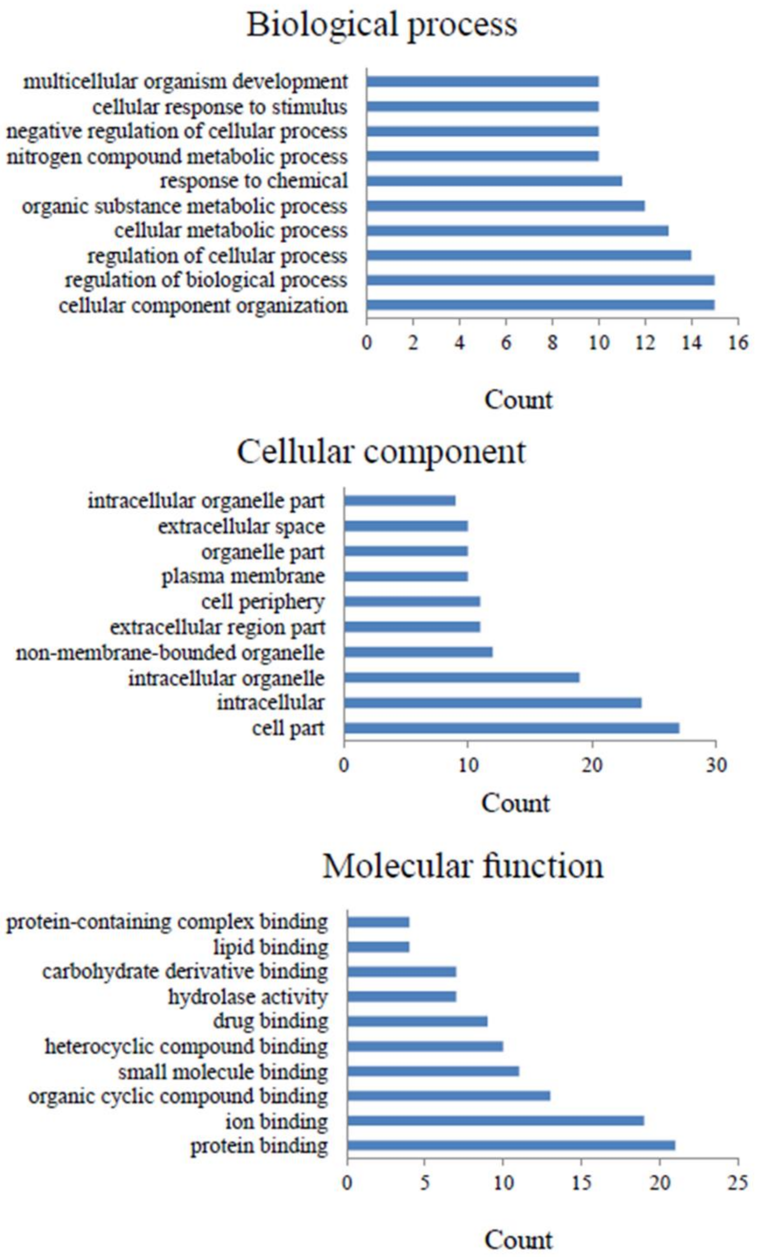

Supplementary Fig. S2. Gene ontology annotation of differentially expressed proteins in the adrenal gland of male L2 strain Taiwan country chickens after acute heat stress.

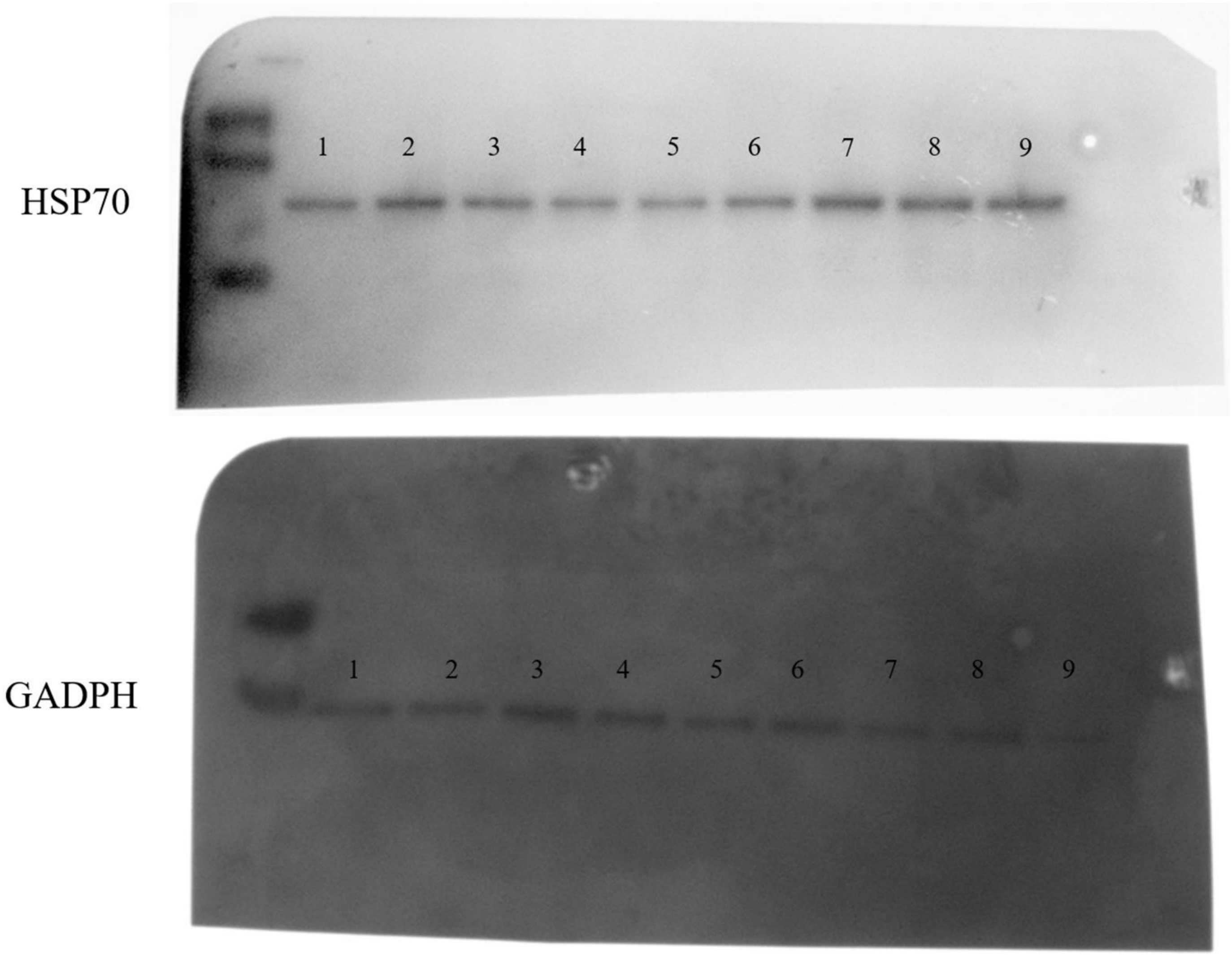

Supplementary Fig. S3. The raw profiles of western blot analysis. HSP70, heat shock protein 70; GADPH, glyceraldehyde-3-phosphate dehydrogenase. Column 1-3, control group; Column 4-6, resistant group; Column 7-9, susceptible group.

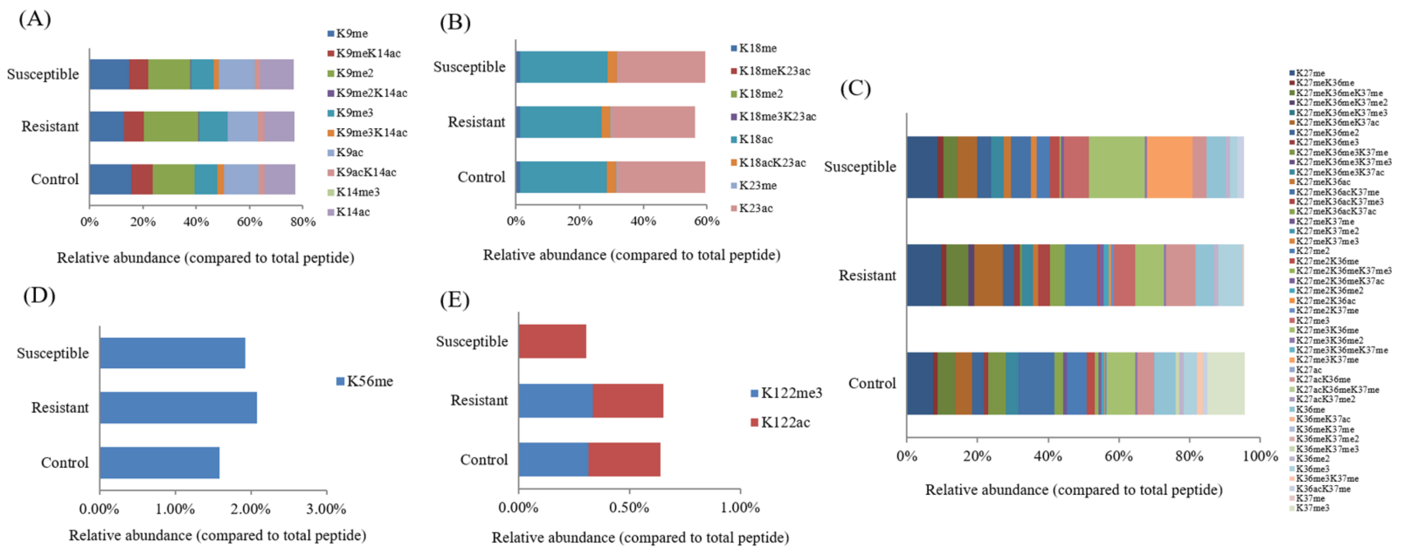

Supplementary Fig. S4. Relative quantification of histone H3 peptide. The relative abundance was estimated using the expression of all peptides covered by these PTMs as 100% (the relative percentage of unmodified peptides is not shown). (A) Peptide KSTGGKAPR (amino acid started at 9th and ended at 17th). (B) Peptide KQLATKAAR (amino acid started at 18th and ended at 26th). (C) Peptide KSAPATGGVKKPHR (amino acid started at 27th and ended at 40th). (D) Peptide YQKSTELLIR (amino acid started at 54th and ended at 63rd). (E) Peptide VTIMPKDIQLAR (amino acid started at 117th and ended at 128th).
